# Mitral valve repair for degenerative mitral regurgitation with Carpentier’s functional classification type II in elderly patients: a single center experience

**DOI:** 10.1186/s13019-024-02578-1

**Published:** 2024-02-09

**Authors:** Masashi Kawamura, Osamu Monta, Shusaku Maeda, Yasushi Tsutsumi

**Affiliations:** Department of Cardiovascular Surgery, Fukui CardioVascular Center, Shinbo 2-228, Fukui City, Fukui Prefecture 910-0833 Japan

## Abstract

**Objective:**

Mitral valve (MV) repair for Carpentier functional classification Type II (C-II) mitral regurgitation (MR) is widely accepted because of its efficacy. It is unclear whether MV repair has the same benefits in elderly patients as in younger patients because of their lower life expectancy. Herein, we examined the midterm results of MV repair for C-II mitral regurgitation, especially in patients aged ≧70 years.

**Method:**

A retrospective review was performed on 176 patients who underwent MV repair for C-II mitral regurgitation with a median age of 65 years; 55 (31%) patients were ≧70 years, and 124 were male (71%). Lesions of the mitral valve were isolated from the anterior leaflet (48 patients), posterior leaflet (113 patients), and both leaflets (15 patients), and included seven patients with Barlow’s disease. We compared the outcomes between patients aged ≧70 years (≧70 years; median age, 76 years) and those aged < 70 years (median age, 60 years).

**Results:**

In terms of the durability of MV repair in elderly patients, there were no significant differences in the rates of freedom from reoperation or MR recurrence at 5 years between patients aged < 70 years and those aged ≧70 years (reoperation:98% in < 70 years versus 89% in ≧70 years; *P* = 0.4053; MR recurrence:95% in < 70 years versus 81% in ≧70 years; *P* = 0.095). The mitral valve complexity was divided into two grades: Simple (isolated posterior mitral lesion) and Complex (isolated anterior lesion or both lesions). In patients aged < 70 years, there was no significant difference in the rate of freedom from MR recurrence at 5 years between the Simple and Complex groups (96% vs. 91%; *P* = 0.1029). In contrast, in patients aged ≧70 years, the MR recurrence rate at 3 years in Complex was significantly higher in the Complex group than in the Simple (100% vs. 80%; *P* = 0.0265).

**Conclusions:**

We studied the outcomes of MV repair for C-II in MR. In elderly patients, MR recurrence was higher in complex lesions than in simple lesions. MV replacement may be considered for elderly patients with complex mitral valve lesions, if appropriately selected.

**Supplementary Information:**

The online version contains supplementary material available at 10.1186/s13019-024-02578-1.

## Introduction

Mitral valve (MV) repair for degenerative mitral disease is widely accepted because of its effectiveness in terms of short- and long-term outcomes [1–3]. MV repair for isolated primary mitral regurgitation (MR) has been performed with increased frequency in the current era, reflecting recent American Heart Association/American College of Cardiology (ACC/AHA) and European Society of Cardiology (ESC) recommendations [4, 5]. However, the benefits of MV repair are still uncertain in some populations, such as older patients and those with specific etiologies. It is unclear whether MV repair has the same benefits in elderly patients as in younger patients because of their lower life expectancy. In MV repair, a return to cardiopulmonary bypass for inadequate MV repair is necessary, and older patients who tend to have comorbidities do not tolerate longer cardiac arrest times or more invasiveness. The durability after MV repair in older patients is another concern. In general, there is a risk of early recurrence of MR after MV repair [6–8], which is especially problematic in elderly patients considering the indication for repeat MV surgery if early recurrence of MR occurs. In the case of recurrent MR in elderly patients, a study of transcatheter edge-to-edge repair in an elderly patient population described that patients with recurrent MR were more likely to experience hospitalization due to heart failure or heart failure symptoms with New York Heart Association scale III or IV, and tended to have a lower survival rate [9]. As described, the efficacy of MV repair in older patients has not been fully investigated. Given these factors, MV replacement with a bioprosthetic valve is still considered an effective procedure for elderly patients [10].

Herein, we review our experience with MV repair for patients with degenerative MR of Carpentier’s functional classification Type II (C-II) and investigate the mid-term clinical results. We also compared the outcomes in patients aged 70 years with those in younger patients to elucidate the effectiveness of MV repair in elderly patients.

## Methods

### Patients

We retrospectively evaluated 176 patients who underwent successful MV repair for C-II MR between July 2010 and December 2021 at our institution. Seventeen patients were excluded because they initially underwent MV repair but were converted to MV replacement because of uncontrolled MR (MV repair rate was 91.2%; 176/193 patients). The current study was approved by the institutional review board of Fukui Cardiovascular Center (2023–41). The requirement for informed consent was waived due to the retrospective nature of this study. Clinical data were retrospectively collected from patient records. All the definitions of the clinical variables were described in the (Additional file 2: supplemental file). Patients with MR of other etiologies, such as rheumatic disease, infection, or secondary causes, were excluded. Procedures concomitant with tricuspid annuloplasty, surgical ablation for atrial fibrillation, left atrial appendage resection, coronary artery bypass grafting, aortic valve replacement, and atrial septal defects were included. Regarding degenerative lesions of the mitral valve, isolated anterior leaflet prolapse (AL) was observed in 48 patients, isolated posterior leaflet prolapse (PL) in 113 patients, and bilateral leaflet prolapse (BL) was observed in 15 patients, including seven with Barlow’s disease. We defined isolated PL lesions as Simple and others, including isolated AL or both lesions, as Complex.

After mitral valve surgery, all patients received warfarin sodium during the first 3 months of sinus rhythm and permanently in atrial fibrillation. Patients were followed-up at our institution or by a cardiologist. Ten patients were lost follow-up during the study period. The median follow-up period was 3.2 years (interquartile range 1.3–6.3 years). Adverse events were reported according to the guidelines [11]. Echocardiography was performed preoperatively, approximately one week after surgery, and during the follow-up period. All studies were assessed by cardiologists at our institution or the referring cardiologists. MR findings were graded as none, trivial, mild, moderate, or severe based on the guidelines of the American Society of Echocardiography [12]. MR recurrence was defined as an MR grade above than moderate during the follow-up period. We investigated the midterm outcomes after MV repair in our institution and compared the outcomes between patients aged < 70 years and those aged ≧70 years, especially regarding durability after MV repair.

## Surgical details

Full sternotomy was performed in 147 patients and right mini-thoracotomy in was performed in 29 patients. Cardiopulmonary bypass was established for aortobicaval cannulation under mild hypothermia, and antegrade and retrograde blood cardioplegia were used for full sternotomy. Peripheral artery and vein cannulation under mild hypothermia and antegrade blood cardioplegia were performed in the right mini-thoracotomy. Ever since the introduction of this approach in 2019, it has been the first choice of treatment for patients with C-II MR. Exclusion criteria for the right mini-thoracotomy are severe arteriosclerosis, low cardiac functions, chest deformity, severe pulmonary dysfunction, and past surgical history of the right chest. Annuloplasty using a ring or band was performed in all patients. The most common repair techniques included leaflet resection and suturing for posterior leaflet prolapse and chordal replacement with expanded polytetrafluoroethylene sutures (Gore-Tex sutures; W.L. Gone & Associates, Inc., Flagstaff, AZ, USA) for anterior leaflet prolapse. Mixed repair procedures were performed for bileaflet prolapse. Additional repair techniques, such as folding plication, edge-to-edge repair, and posteromedial commissuroplasty for minor residual leakage, were mainly based on the surgeon’s preference. All patients were evaluated for remnant mitral regurgitation using intraoperative transesophageal echocardiography and were confirmed to have no or trivial mitral regurgitation after cardiopulmonary bypass weaning. When mitral regurgitation was not controlled, the procedure was converted to MV replacement.

## Statistical analyses

Continuous values were expressed as mean ± standard deviation or median and interquartile ranges when their distribution was skewed, and were compared using the Wilcoxon rank-sum test. Categorical variables were summarized as frequencies and percentages, and were compared using the χ^2^test or Fisher’s exact test when fewer than five events were observed in either group. Statistical significance was set less than 0.05. Kaplan–Meier analysis was used to estimate time-related events, including the rates of survival, freedom from reoperation, and freedom from recurrent moderate or severe MR. Comparisons between groups were performed using the log-rank test or generalized Wilcoxon test. All data analyses were performed using JMP software (version 15; SAS Institute, Inc., Cary, NC, USA).

## Results

Baseline characteristics of the study cohort are summarized in Table 1. We compared each factor between patients aged < 70 years and those aged ≧70 years (Table 1). The median age was 60 years- in < 70 years group, and 76 years in ≧70 years group. The percentage of male patients and body surface area were significantly higher in patients aged < 70 years, and the prevalence of chronic atrial fibrillation was significantly higher in patients aged ≧70 years. EuroSCORE II in patients aged ≧70 years was significantly higher than that in patients aged < 70 years. The operative procedures are described in Table 2. Mitral surgery via right mini-thoracotomy was performed in 29 patients (16%), and the minimally invasive approach was significantly more common in patients aged < 70 years. There were no significant differences in major concomitant procedures, including coronary artery bypass grafting or aortic valve replacement, between the groups. Concomitant tricuspid procedures were significantly more common in patients aged ≧70 years. There were no significant differences in cardiopulmonary or aortic cross-clamp times between the groups. Mitral valve lesions and mitral repair techniques are shown in detail in Table 3. There were no significant differences in the location or complexity of mitral valve lesions between the two groups. Resection and suture techniques were more frequently used in patients aged < 70 years, and artificial chordoplasty was more often used in patients aged ≧70 years. This indicated that patients aged ≧70 years had more anterior leaflet lesions. Mitral annuloplasty using a ring or band was performed in all patients. The size of the ring or band used in patients aged ≧70 years was significantly smaller than that used in patients aged < 70 years. The MV repair rate in our institution is shown in the (Additional file 3: supplemental table).

**Table 1:** Baseline Characteristics of Study Cohort

Characteristics	Overall (n = 176)	Total Cohort (n = 176)		
		< 70 years (n = 121)	≧70 years (n = 55)	*p* Value
Age, median (IQR), years	65 (56–71)	60 (50.5–65)	76(72–78)	< 0.0001
Male	124 (70.5)	92 (76.0)	32 (58.2)	0.0206
Body mass index, mean (SD)	22.4 (3.8)	23.4 (3.8)	20.3 (2.9)	< 0.0001
Body surface area, mean (SD), m^2^	1.59 (0.22)	1.67 (0.19)	1.43 (0.16)	< 0.0001
Diabetes mellitus	18 (10.2)	12 (9.9)	6 (10.9)	0.7953
Dyslipidemia	60 (34.1)	40 (33.1)	20 (36.4)	0.7323
Hypertension	94 (53.4)	59 (48.8)	35 (63.6)	0.0746
Chronic lung disease	6 (3.4)	2 (1.7)	4 (7.2)	0.0773
Chronic renal failure	3 (1.7)	1 (0.8)	2 (3.6)	0.2305
Peripheral vascular disease	7 (4.0)	4 (3.3)	3 (5.5)	0.6685
Cerebrovascular disease	5 (2.8)	1 (0.8)	4 (7.3)	0.0337
History of heart failure	45 (25.6)	26 (21.5)	19 (34.5)	0.0925
Atrial fibrillation	71 (40.3)	45 (37.2)	26 (47.3)	0.2465
Chronic	46 (26.1)	26 (21.5)	20 (36.4)	0.0431
Paroxysmal	25 (14.2)	19 (15.7)	6 (10.9)	0.4891
Previous myocardial infarction	4 (2.3)	2 (1.7)	2 (3.6)	0.5900
EF, median (IQR)	59 (55–61)	58 (55–60)	60 (53–62)	0.4023
Coronary artery disease	21 (11.9)	11 (9.1)	10 (18.2)	0.1298
NYHA functional class III-IV	88(50.0)	55(45.5)	33(60.0)	0.1842
MR grade				0.7291
moderate	66(37.5)	47(38.8)	19(34.5)	
severe	110(62.5)	74(61.2)	36(65.5)	
EuroSCORE II, median (IQR), %	1.4(0.9–2.3)	1.1(0.8–1.6)	2.4(1.7–4.2)	< 0.0001

Values are n (%) unless otherwise indicated

SD = standard deviation; EF = ejection fraction; IQR = interquartile range; NYHA = New York Heart Association; MR = mitral regurgitation

**Table 2:** Operative Characteristics

Characteristics	Overall (n = 176)	Total Cohort (n = 176)		
		< 70 years (n = 121)	≧70 years (n = 55)	*p* Value
Surgical approach				0.0287
Median sternotomy	147 (83.5)	96 (79.3)	51 (92.7)	
Right mini-thoracotomy	29 (16.5)	25 (20.7)	4 (7.3)	
CPB time, minutes, median (IQR)	151 (120–184)	150 (119–184)	154 (123–184)	0.6527
Aortic cross clamp time, minutes, median (IQR)	97 (71–124)	95 (69–120)	101 (79–128)	0.7073
Tricuspid procedure	86 (48.9)	43 (35.5)	43 (78.2)	< 0.0001
Atrial fibrillation surgery	63 (35.8)	44 (36.4)	19 (34.5)	0.8659
Maze procedure	55 (31.3)	37 (30.6)	18 (32.7)	0.8610
PV isolation	8 (4.5)	7 (5.8)	1 (1.8)	0.4379
Left atrial appendage resection	71 (40.3)	46 (38.0)	25 (45.5)	0.4080
CABG	18 (10.2)	9 (7.4)	9 (16.4)	0.1045
Aortic valve replacement	10 (5.7)	5 (4.1)	5 (9.1)	0.2038
ASD closure	3 (1.7)	2 (1.7)	2 (3.6)	0.4305

Values are n (%) unless otherwise indicated

CPB = cardiopulmonary bypass; IQR = interquartile range; PV = pulmonary vein; CABG = coronary artery bypass grafting; ASD = atrial septal defect

**Table 3:** Mitral Valve Lesion and Reconstructive Techniques in Details

Characteristics	Overall (n = 176)	Total Cohort (n = 176)		
		< 70 years (n = 121)	≧70 years (n = 55)	*p* Value
Mitral valve lesions				
Isolated anterior leaflet prolapse	48 (27.2)	29 (24.0)	19 (34.5)	0.1494
Isolated posterior leaflet prolapse	113 (64.2)	82 (67.8)	31 (56.4)	0.1751
Bileaflet prolapse	15 (8.5)	11 (9.1)	4 (7.3)	0.8725
Barlow’s disease	7 (4.0)	6 (5.0)	1 (1.8)	0.4367
Reconstructive technique				
Resection & suture	112 (63.6)	84 (69.4)	28 (50.9)	0.0274
Artificial chordoplasty	54 (30.7)	31 (25.6)	23 (41.8)	0.0355
Folding plication	10 (5.7)	6 (5.0)	4 (7.3)	0.5059
Edge-to-edge repair	8 (4.5)	6 (5.0)	2 (3.6)	0.6906
Posteromedial commisuroplasty	4 (2.3)	4 (3.3)	0 (0)	0.3109
Mitral annuloplasty	176(100)	121(100)	55(100)	
Ring	77(44)	49(40)	28(51)	0.1968
Band	99(56)	72(60)	27(49)	0.1968
Size of ring or band, median (IQR)	30(28–30)	30(28–30)	28(28–30)	0.0271

Values are n (%) unless otherwise indicated

IQR = interquartile range

**Fig. 1:**
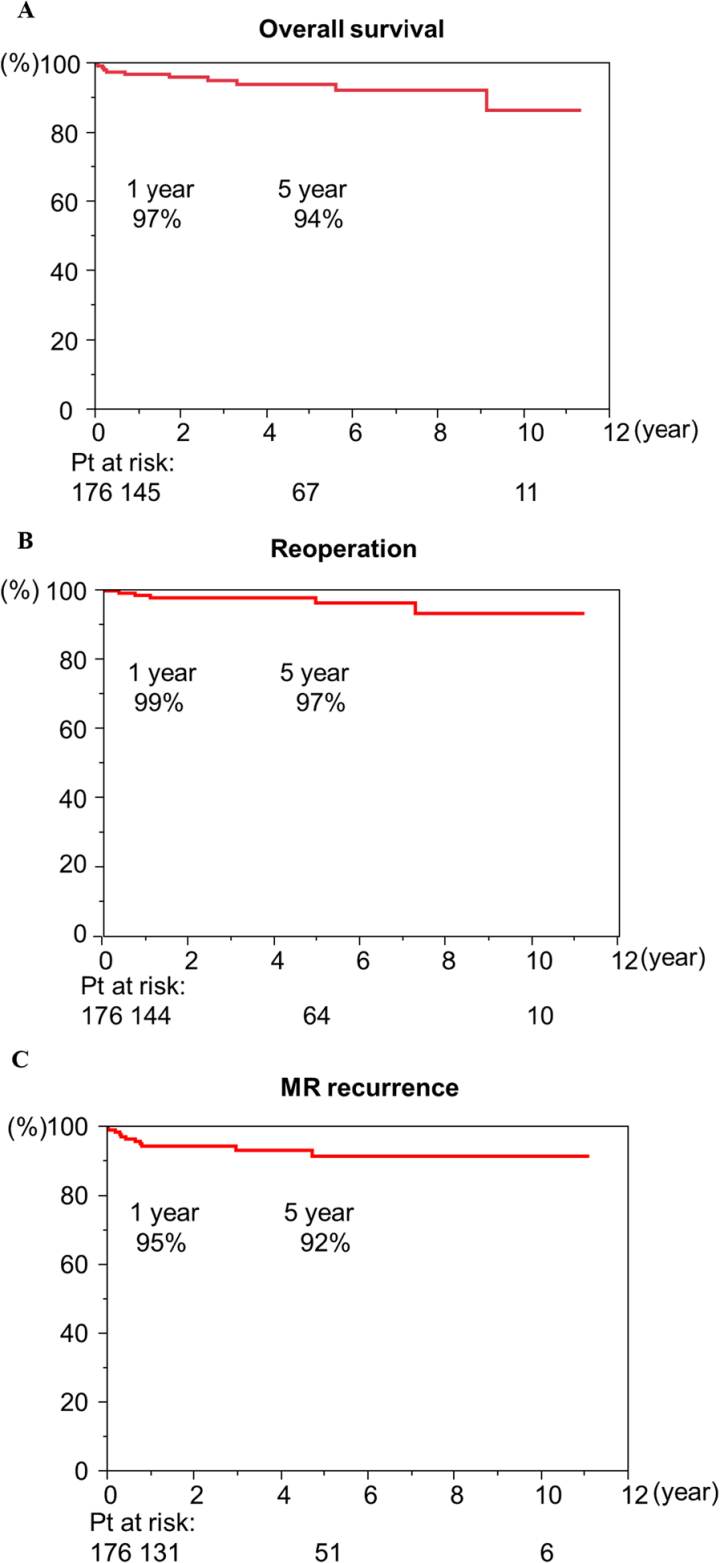
Kaplan–Meier analysis of total cohort. Cumulative survival rate **A**, reoperation-free rate (B), and MR recurrence-free rate (C) were shown in Fig. 1

**Fig. 2:**
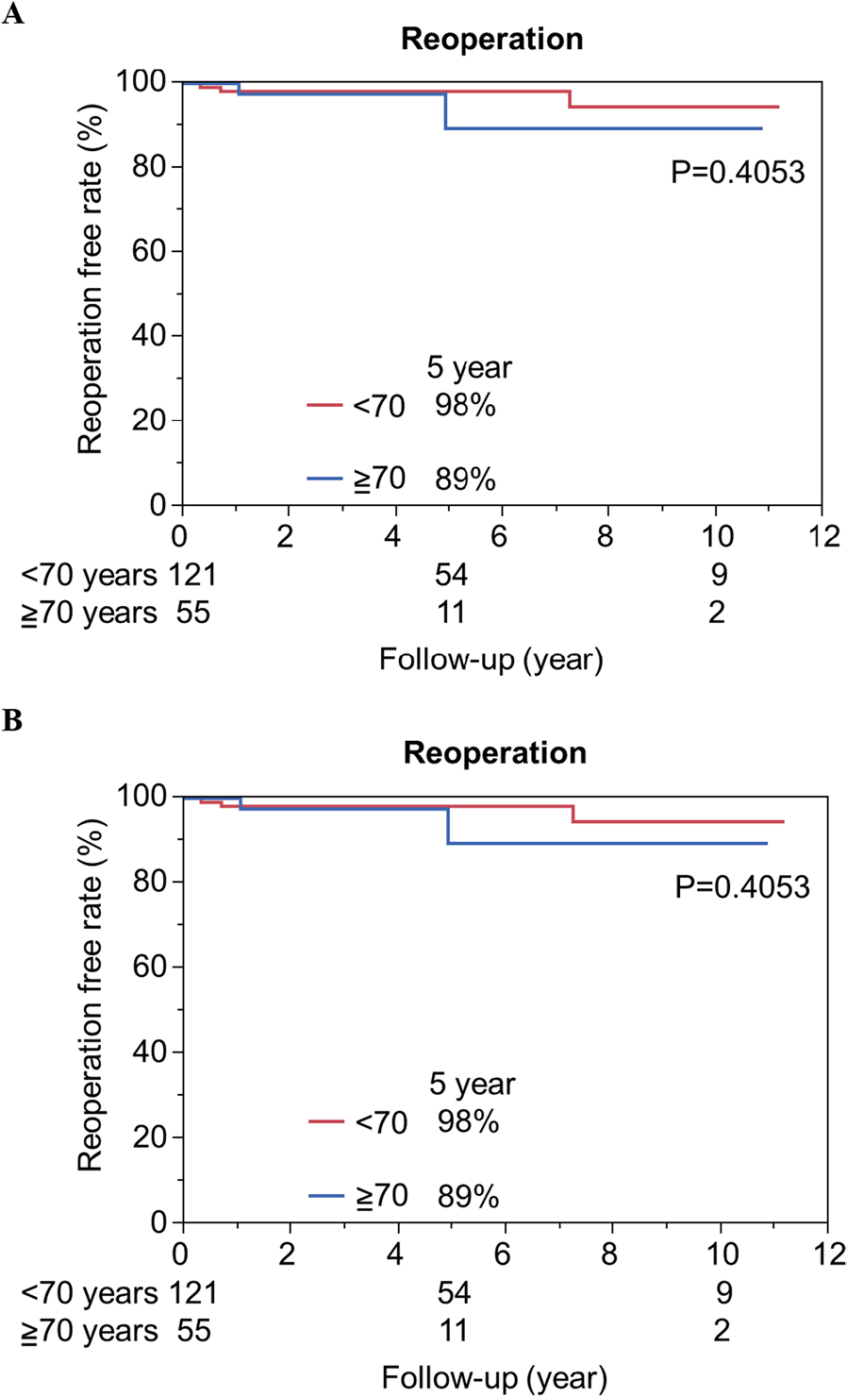
The durability of MV repair; < 70 years vs. ≧70 years. Reoperation-free rate **A** and MR recurrence-free rate **B** were shown in Fig. 2

**Fig. 3:**
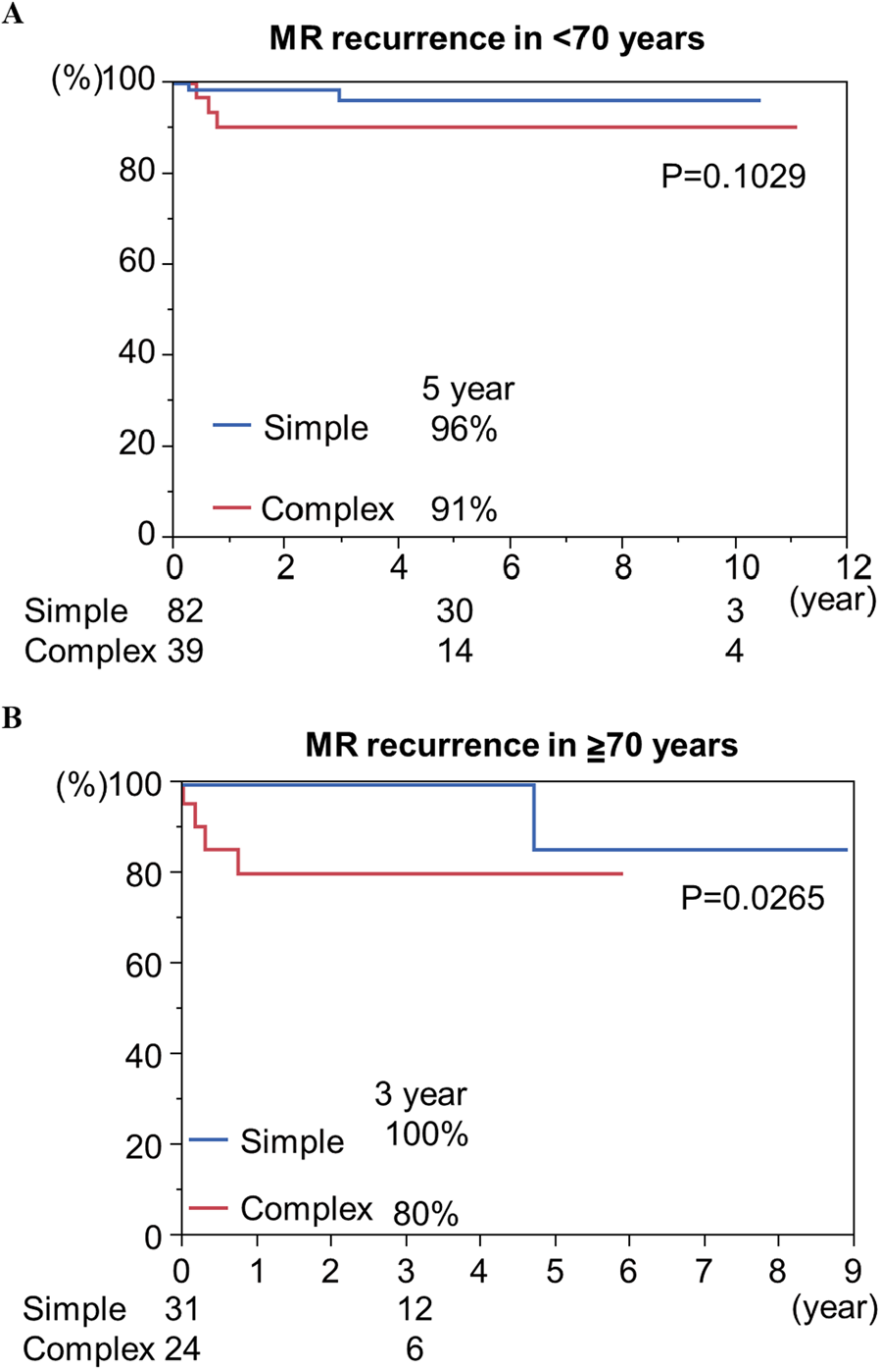
The durability of MV repair in terms of mitral lesion complexity. MR recurrence-free rate in < 70 years **A** and in ≧70 years **B** were shown in Fig. 3

In all the cohorts, the 1- and 5-year cumulative survival rates were 97%, and 94%, respectively (Fig. 1A). The 1-, and 5-year reoperation free rates were 99% and 97%, respectively (Fig. 1B). The rates of freedom from MR recurrence at 1 and 5 years were 95% and 92%, respectively (Fig. 1C). In terms of the durability of MV repair in elderly patients, there were no significant differences in the rates of freedom from reoperation or MR recurrence at 5 years in patients aged < 70 years versus those aged ≧70 years (Fig. 2A, B) (reoperation:98% in < 70 years versus 89% in ≧70 years; P = 0.4053; MR recurrence:95% in < 70 years versus81% in > 70 years; P = 0.0955). We investigated the influence of mitral lesion complexity on the durability in each group. The mitral valve complexity was divided into two grades: Simple, which included isolated posterior mitral lesions, and Complex, which included isolated AL or both lesions. In patients aged < 70 years (N = 121), there was no significant difference in the rate of freedom from MR recurrence at 5 years between the Simple and Complex groups, as shown in Fig. 3A (96% in the Simple group vs. 91% in the Complex group; P = 0.1029). In contrast, in patients aged ≧70 years, the MR recurrence rate at 3 years was significantly higher in the complex group than in the Simple group (Fig. 3B) (100% in the simple group versus 80% in the complex group; *P* = 0.0265). We also investigated the influence of operative characteristics, including surgical approach, concomitant tricuspid procedure, and mitral annuloplasty (ring or band) on the durability of MV repair within each age group. These factors did not demonstrate significant differences, as shown in the (Additional file 1: Supplemental figure).

## Discussion

At our institution, the midterm outcomes of overall survival, reoperation, and MR recurrence after mitral valve repair for degenerative MR were satisfactory. Although there were no significant differences in reoperation or MR recurrence between patients aged < 70 years and those aged ≧70 years, the elderly patients with complex mitral valve lesions had a significantly higher rate of MR recurrence than those with simple mitral valve lesions.

The durability after MV repair is particularly important in elderly patients. Javadikasgari et al. reported that the long-term durability of MV repair for simple diseases (isolated posterior prolapse) was significantly better than that for complex diseases, such as anterior or bileaflet prolapse [13]. Suri et al. analyzed the rate of MR recurrence after MV repair according to the localization of prolapse and found that patients with isolated posterior leaflet prolapse exhibited significantly less MR recurrence compared with isolated anterior leaflet or bileaflet prolapse [14]. These studies demonstrate that MV pathology affects the durability of MV repair and support the results of the present study. Kawajiri et al. demonstrated excellent outcomes of MV repair in patients aged 75 years in an experienced and high-volume center; operative mortality was 1.2% and the reoperation-free rate at 10 years was 3.2%;they concluded that MV repair was preferred for elderly patients [15]. The ratio of isolated posterior prolapse in the entire cohort was 78.4%, which was relatively high compared to that of the present study and other previous studies [3, 7, 14] (approximately 50–60%). This factor may influence early and late outcomes of MV repair in elderly patients.

It is well-known that there is a spectrum of degenerative MV disease ranging from fibroelastic deficiency (FED) to Barlow’s disease. FED has thin transparent leaflets, and patients with FED are typically aged > 60 years [16]. In contrast, Barlow’s disease is characterized by diffuse excess leaflet tissue with myxomatous changes, and patients with Barlow’s disease are generally younger [16]. In the current study, patients aged ≧70 years might have a higher prevalence of fibroelastic deficiency that is characterized by thin transparent leaflets with less tissue. The histological fragility of the MV leaflets can affect their durability after MV repair. In addition, patients aged ≧70 years included more chronic atrial fibrillation and coronary artery disease even though those were not statistically significant compared with patients aged < 70 years. Ischemic and atrial functional MR are other important etiologies of MR, and it has been reported that the recurrence rates of moderate or severe MR after MV repair for these etiologies are high. The MR recurrence rate of MV repair for ischemic MR was 58.8% within 2 years [17] and that for atrial functional MR was 16.8% at 5 years [18]. These ischemic and/or atrial contributions to MR recurrence may also be observed in older patients.

MV replacement is another effective treatment option for elderly patients with degenerative MR. Recent reports have demonstrated excellent early and long-term outcomes with sufficient durability of artificial valves at the mitral position [19, 20]; however, rare but deadly complications of left ventricular dehiscence and attenuated cardiac function occur in the early phase after MV replacement [21]. It remains controversial whether MV repair or replacement is better for elderly patients with degenerative MR. In previous studies on MV repair vs. MV replacement in elderly patients, a study using a national database demonstrated that the early results of MV repair were significantly better than those of MV replacement [22]. However, in this study, the patient characteristics differed between the groups, and there were more patients with older age, diabetes mellitus, and chronic lung disease in the MV replacement group. In contrast, the early and late outcomes of MV repair and replacement in elderly patients were equivalent in studies using propensity score matching analysis [23, 24]. Ko et al. reported that not the type of surgery, MV repair or MV replacement, but higher age, diabetes, and left ventricular dysfunction were significant risk factors for late outcomes after MV surgery in elderly patients [24]. They also demonstrated in a sub-analysis of elderly patients with degenerative disease who were almost the same age as in our study that there was no difference in long-term survival between MV repair for simple lesions and MV replacement for complex lesions. The data are informative, although the surgical approach, MV repair technique, and patient comorbidities were different from those in our study [24]. Elderly patients who undergo MV replacement can obtain the same benefits as those who undergo MV repair if appropriately selected and indicated.

Recent advances in computer science are remarkable, and the application of this technology in medicine, including mitral valve surgery, has begun. A few recent studies have used machine learning algorithms to predict the repairability and durability of MV repair in precision medicine [25, 26]. Penso et al. created a model to predict MV repair success using a machine learning method and applied a dataset that included MV prolapse complexity, in addition to clinical and procedural characteristics, to increase the accuracy of the predictive model [25]. This approach for patient-specific prediction of MV repair is promising. Simultaneously, the risk of early mortality from MV replacement could be predicted. The use of this technology could predict patients with low repairability and durability in MV repair and low early mortality in MV replacement; these patients, particularly elderly patients, could be recommended to undergo MV replacement. In addition, transcatheter edge-to-edge repair has emerged as a less invasive option for MV surgery. This procedure has demonstrated excellent outcomes in patients with degenerative MR [27]. Recently, patients with degenerative MR, especially elderly patients, have received more treatment options. This technology of computer science may potentially contribute to appropriate patient selection regarding the type of MV surgery and may also improve the outcomes after MV surgery.

Our study had some limitations. This was a retrospective observational study and single-center analysis of patients who underwent MV repair for MR with C-II. The additional technique of mitral repair and the selection of an annuloplasty ring or band were based on the surgeon’s preference, which may have affected the outcomes, including MR recurrence or reoperation after MV repair.

In conclusion, we assessed the outcomes of MV repair for degenerative MR at our institution and found that the mid-term results were acceptable. Elderly patients with complex lesions tended to have more recurrent MR than those with simple lesions. MV replacement may be considered for elderly patients with complex lesions, if patients are appropriately selected.

### Supplementary Information

**Additional file 1**: Surgical approach.

**Additional file 2**: Definitions of the clinical variables.

**Additional file 3**: MV repair rate.

## Data Availability

The data of this study will be provided to the editor of the journal if it is needed.
