# Supplementary material for: Mitral valve repair for degenerative mitral regurgitation with Carpentier’s functional classification type II in elderly patients: a single center experience
Source: J Cardiothorac Surg. 2024 Feb 9;19:75. doi: 10.1186/s13019-024-02578-1 (PMC10854023; doi:10.1186/s13019-024-02578-1)
Supplement: Supplementary file 1 — Additional file 1: Surgical approach. [file 13019_2024_2578_MOESM1_ESM.docx]

**Supplemental figure**

**Surgical approach**

Full sternotomy (F)

Right mini-thoracotomy (R)

**
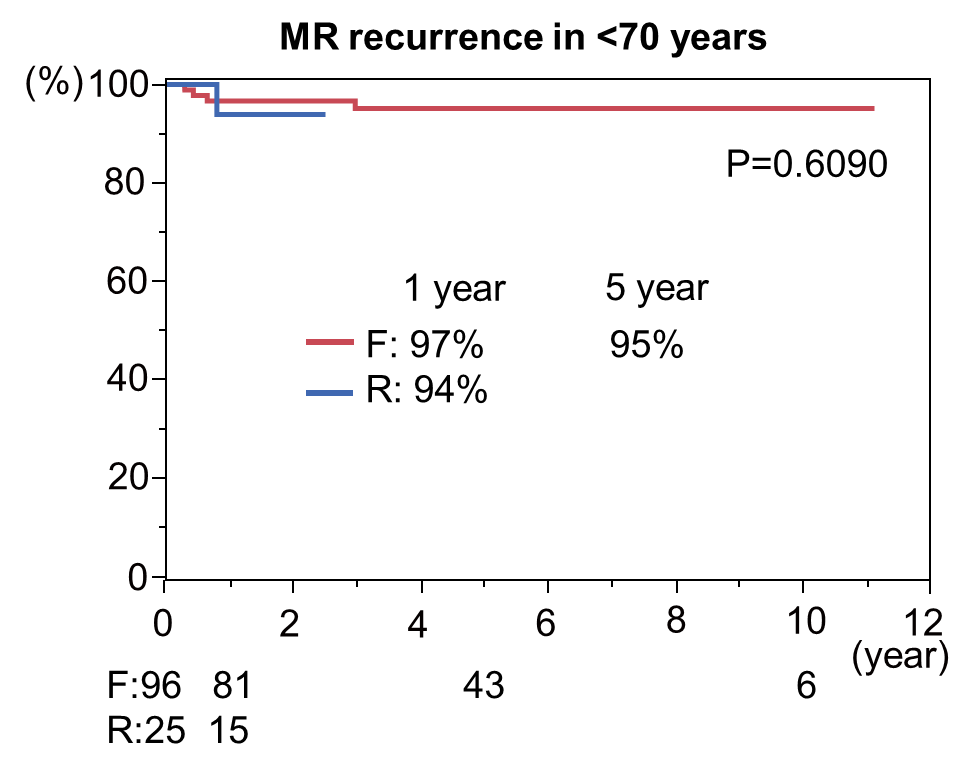
**

**
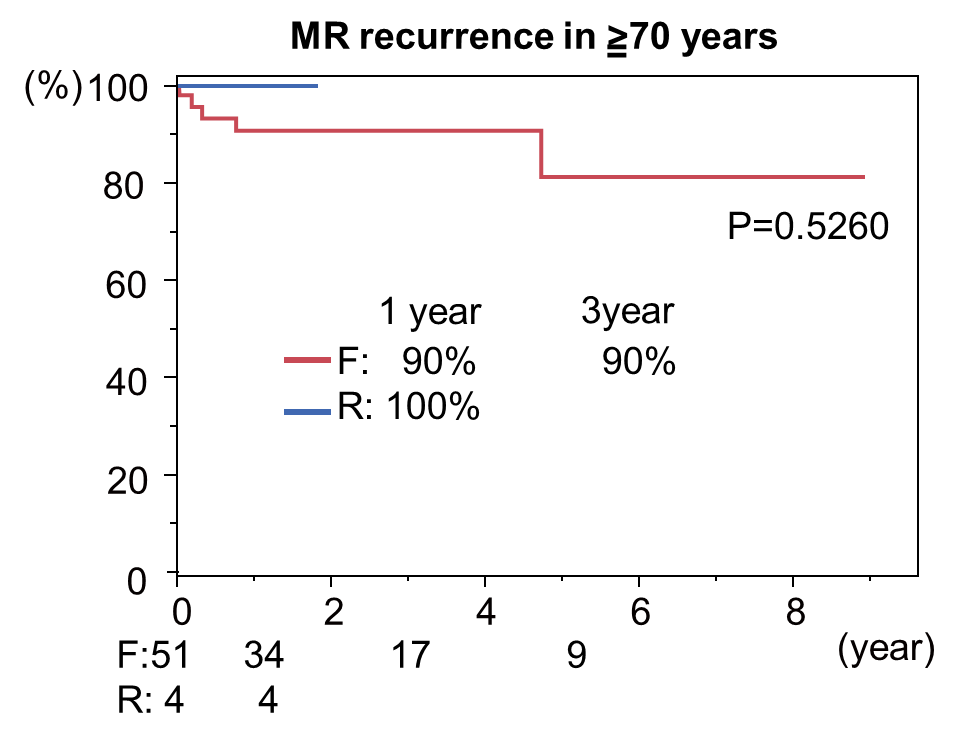
**

**Concomitant procedure**

**
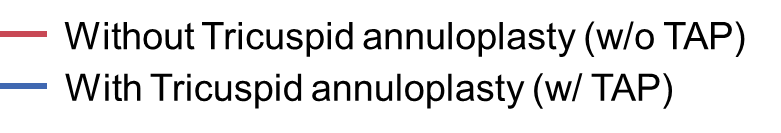
**

**
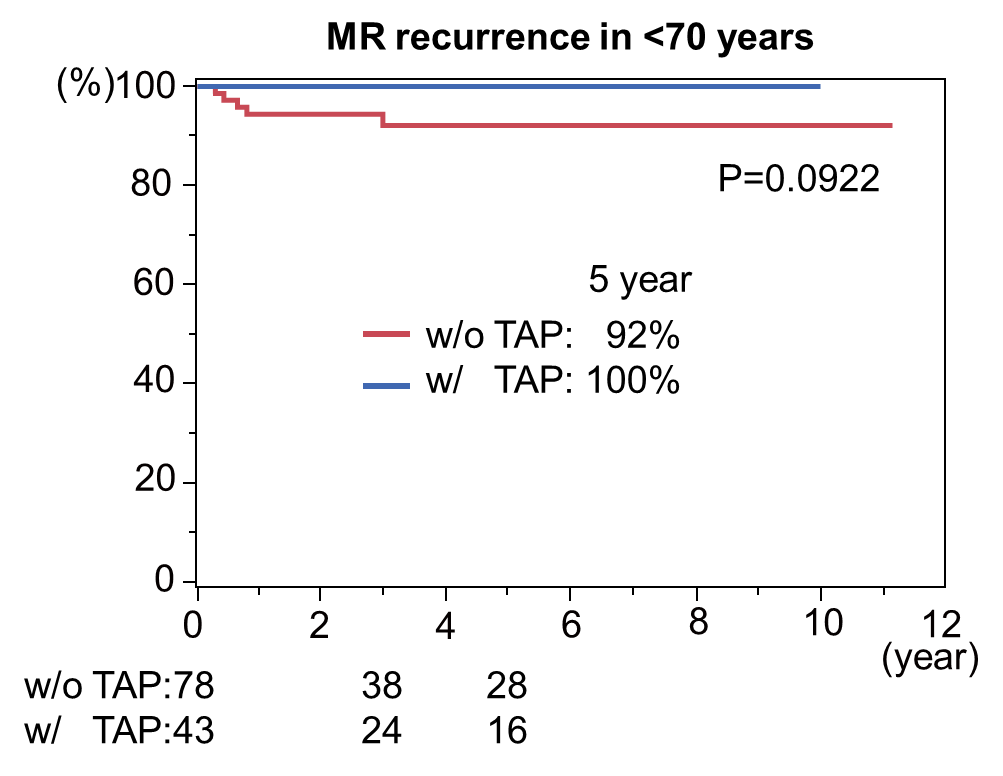
**

**
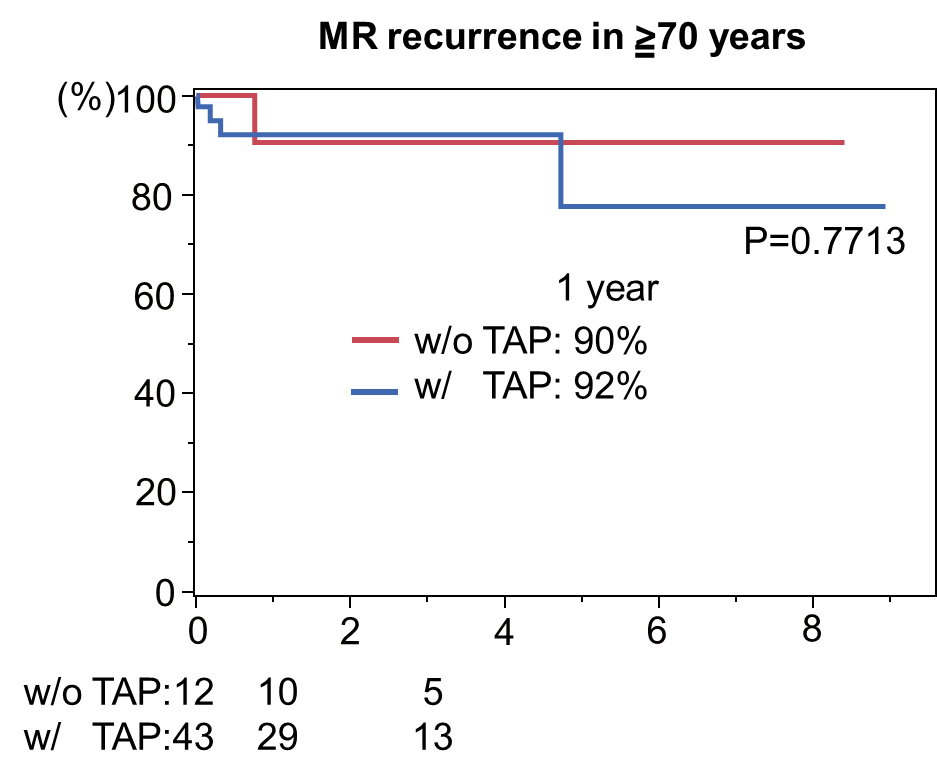
**

**Mitral annuloplasty**

**
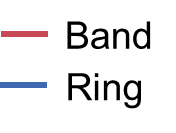
**

**
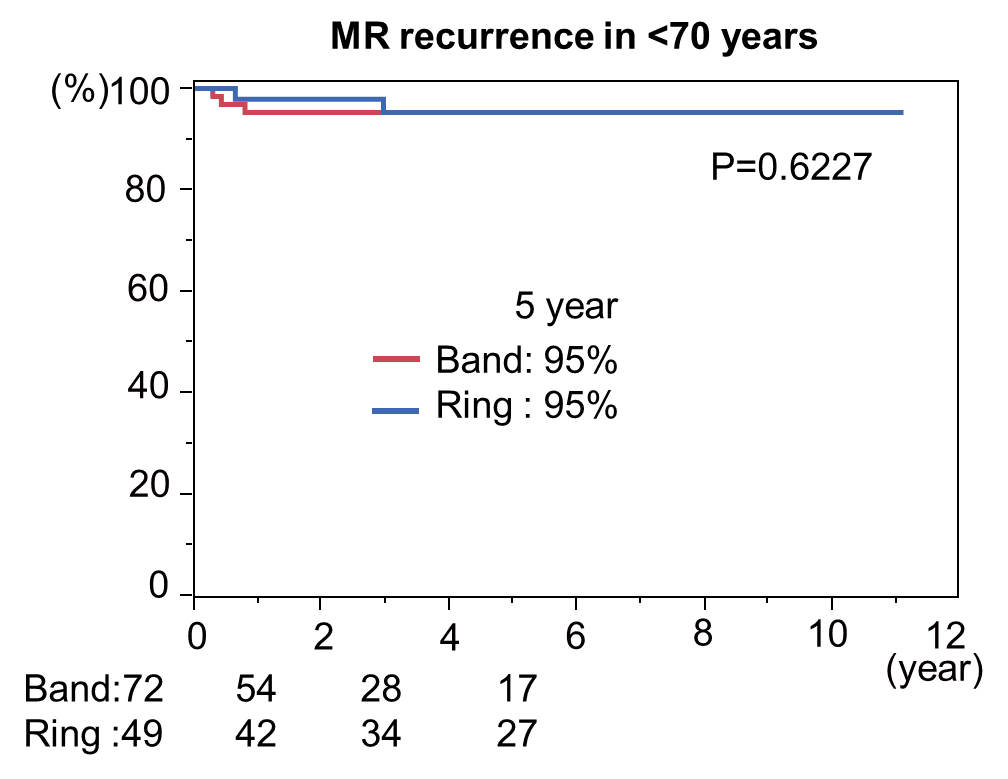
**

**
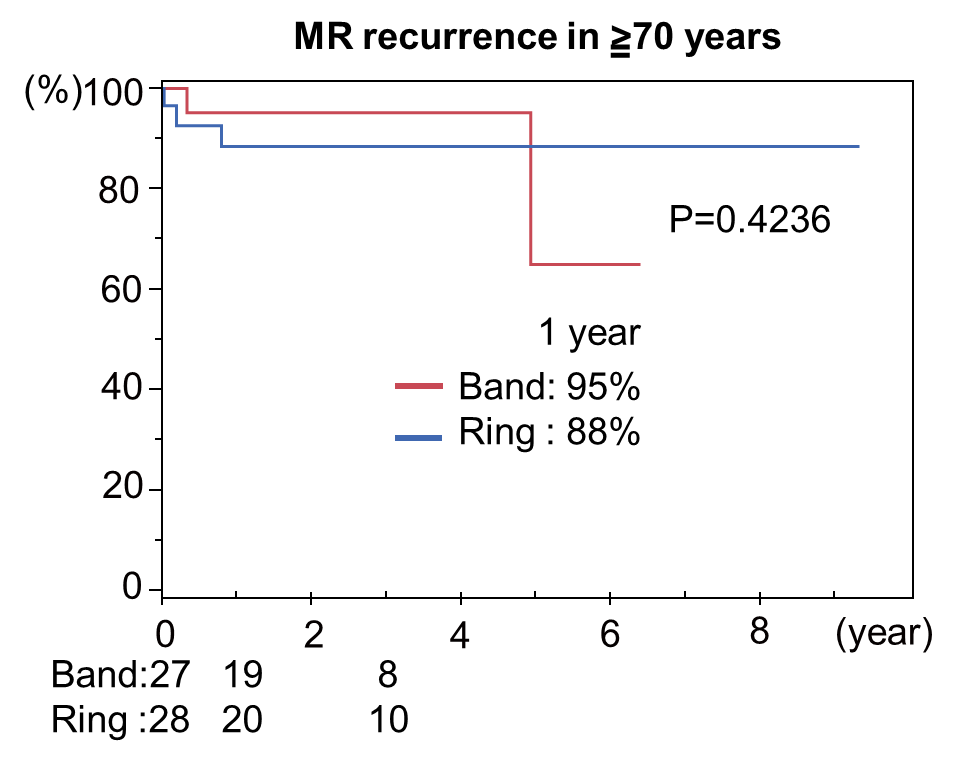
**
