# Supplementary material for: Mitral valve repair for degenerative mitral regurgitation with Carpentier’s functional classification type II in elderly patients: a single center experience
Source: J Cardiothorac Surg. 2024 Feb 9;19:75. doi: 10.1186/s13019-024-02578-1 (PMC10854023; doi:10.1186/s13019-024-02578-1)
Supplement: Supplementary file 2 — Additional file 2: Definitions of the clinical variables. [file 13019_2024_2578_MOESM2_ESM.docx]

All the definitions of the clinical variables were described as below.

| Diabetes mellitus | diabetes mellitus treated with oral medication |
| --- | --- |
| Dyslipidemia | dyslipidemia treated with oral medication |
| Hypertension | hypertension treated with oral medication |
| Chronic lung disease | long-term use of bronchodilators or steroids for lung disease |
| Chronic renal failure | creatinine clearance <30ml/min |
| Peripheral vascular disease | one or more of claudication, carotid occlusion or 50% stenosis, previous intervention on the limb arteries or carotids |
| Cerebrovascular disease: | old cerebral infarction |
| History of heart failure admission | History of admission due to acute heart failure |
| Old myocardial infarction | electrocardiographic finding of pathologic Q waves |
| Coronary artery disease | single vessel or more of coronary artery disease or previous percutaneous coronary intervention |
