# Supplementary material for: Mitral valve repair for degenerative mitral regurgitation with Carpentier’s functional classification type II in elderly patients: a single center experience
Source: J Cardiothorac Surg. 2024 Feb 9;19:75. doi: 10.1186/s13019-024-02578-1 (PMC10854023; doi:10.1186/s13019-024-02578-1)
Supplement: Supplementary file 3 — Additional file 3: MV repair rate. [file 13019_2024_2578_MOESM3_ESM.docx]

Supplemental Table.

|  |  | Total (n=193) | | | |
| --- | --- | --- | --- | --- | --- |
| Characteristics | Overall (n=193) | | <70 years (n=131) | ≧70 years (n=62) | p Value |
| Intraoperative conversion from MV repair  to MV replacement | 17(8.8) | | 10(7.6) | 7(11.3) | 0.403 |
|  |  | |  |  |  |
| MV repair rate, % | 91.2 | | 92.4 | 88.7 |  |
|  |  | |  |  |  |

Values are n (%) unless otherwise indicated.

MV=mitral valve.
